# Lithium Ascorbate as a Promising Neuroprotector: Fundamental and Experimental Studies of an Organic Lithium Salt

**DOI:** 10.3390/molecules27072253

**Published:** 2022-03-30

**Authors:** Ivan Yu. Torshin, Olga A. Gromova, Konstantin S. Ostrenko, Marina V. Filimonova, Irina V. Gogoleva, Vladimir I. Demidov, Alla G. Kalacheva

**Affiliations:** 1 Federal Research Center “Computer Science and Control” of Russian Academy of Sciences, 119333 Moscow, Russia; tiy135@yahoo.com; 2 All-Russian Research Institute of Physiology, Biochemistry and Animal Nutrition, 249013 Borovsk, Russia; ostrenkoks@gmail.com; 3 A. Tsyba Medical Radiological Research Center, 249036 Obninsk, Russia; vladimirovna.fil@gmail.com; 4 Ivanovo State Medical Academy, 153012 Ivanovo, Russia; gogolev04@yandex.ru (I.V.G.); 13vid@mail.ru (V.I.D.); alla_kalacheva@mail.ru (A.G.K.)

**Keywords:** lithium therapy, neurocytology, toxicology, neuroprotection, chemoinformatics, big data

## Abstract

Given the observable toxicity of lithium carbonate, neuropharmacology requires effective and non-toxic lithium salts. In particular, these salts can be employed as neuroprotective agents since lithium ions demonstrate neuroprotective properties through inhibition of glycogen synthetase kinase-3β and other target proteins, increasing concentrations of endogenous neurotrofic factors. The results of theoretical and experimental studies of organic lithium salts presented here indicate their potential as neuroprotectors. Chemoreactomic modeling of lithium salts made it possible to select lithium ascorbate as a suitable candidate for further research. A neurocytological study on cerebellar granular neurons in culture under conditions of moderate glutamate stress showed that lithium ascorbate was more effective in supporting neuronal survival than chloride or carbonate, i.e., inorganic lithium salts. Biodistribution studies indicated accumulation of lithium ions in a sort of “depot”, potentially consisting of the brain, aorta, and femur. Lithium ascorbate is characterized by extremely low acute and chronic toxicity (LD50 > 5000 mg/kg) and also shows a moderate antitumor effect when used in doses studied (5 or 10 mg/kg). Studies on the model of alcohol intoxication in rats have shown that intake of lithium ascorbate in doses either 5, 10 or 30 mg/kg did not only reduced brain damage due to ischemia, but also improved the preservation of myelin sheaths of neurons.

## 1. Introduction

Lithium is an essential trace element that exhibits pronounced neuroprotective and neurotrophic effects. Although lithium carbonate has been used in the pharmacotherapy of bipolar disorder for over 60 years [1], this is minimal use of lithium salts. Much more important is the fact that lithium is necessary for the normal functioning of all body systems and, above all, the nervous system [2]. Lithium is involved in the metabolism of simple sugars, lipids, regulation of blood pressure and hematopoiesis, regulation of inflammation, in the homeostasis of neurotransmitters, and, in general, exhibits neurotrophic and neuroprotective effects [3]. Organic lithium salts can enhance the effects of other neuroprotective agents [4].

Lithium ions exert their effects by activating neuroprotective and neurotrophic cell cascades. The mechanisms by which these effects of lithium are mediated include inhibition of glycogen synthetase kinase-3β (GSK-3β), induction of autophagy, inhibition of N-methyl-D-aspartate (NMDA) receptors, and anti-apoptotic effects. GSK-3β is a negative regulator of the Wnt cascade required for axonal growth. Inhibition of GSK-3β by lithiumions promotes activation of the neurotrophic cascade Wnt, accelerates the differentiation of neuronal progenitor cells, stimulates differentiation of astrocytes, myelin synthesis, supports neuronal survival, expression of brain-derived neurotrophic factor (BDNF), and of other neurotrophic factors [5]. Clinical studies show that lithium therapy induces an increase in gray matter volume [6]. It is advisable to use lithium preparations in patients with amyotrophic lateral sclerosis [7]. An interesting direction in developing neuroprotective drugs is related to the studies of organic lithium salts, characterized by low toxicity and high bioavailability [8].

Commonly used lithium carbonate has significant drawbacks, especially when taken for a long time (months) in high doses (grams). First, for the effective and safe use of these lithium carbonate drugs, it is necessary to regularly measure the concentration of lithium in the blood, which is an additional invasive procedure [9]. Secondly, lithium carbonate is characterized by a narrow therapeutic corridor of lithium concentrations in plasma (0.6–1.2 mmol/L). Li+ concentrations of 1.5–2.5 mmol/L are associated with mild toxicity, 2.5–3.5 mmol/L—with severe poisoning, and Li+ concentration in blood plasma exceeding 3.5 mmol/L can be life-threatening [10]. Thirdly, to achieve the desired therapeutic effect in psychiatry, it is necessary to use significant dosages of lithium carbonate (1–3 g/day, in the acute period—up to 9 g/day), which, due to the high toxicity of the salt, sharply reduces its spectrum of clinical applications [11].

Therefore, it is relevant to the search for such anions for the synthesis of lithium salts that (1) maximize the flow of lithium ions into neurons, (2) would be characterized by the most suitable spectrum of pharmacological activities, and (3) would not show toxic effects even with prolonged use.

This work presents the search results for organic anions most suitable for the synthesis of neuroprotective lithium salts. The actual screening and analysis of the pharmacological effects of organic anions were carried out in silico using the latest mathematical apparatus developed at the scientific school of Acad. RAS Yu.I. Zhuravlev (chemoreactomic modeling) and the proprietary software complex [12,13]. Then, a complex of in vitro and in vivo studies were carried out for the selected candidates. This paper presents the results of studies of lithium ascorbate, including neurocytological studies on cerebellar granular neurons in vitro (model of glutamate stress), analysis of biodistribution, acute and chronic toxicity, antitumor effects, and neuroprotective properties in vivo estimated on the model of alcohol intoxication.

## 2. Results

Chemoreactomic modeling made it possible to select lithium ascorbate as the most promising salt for further studies. After the synthesis of the lithium ascorbate its neurocytological study was carried out on the granular neurons of the cerebellum. Animal studies of the lithium ascorbate included assessments of lithium biodistribution, studies of acute and chronic toxicity, antitumor properties, adaptogenic and neuroprotective effects.

### 2.1. Results of Chemoreactomic Modeling of Lithium Salts

Chemoreactomic screening of lithium salts was performed in three stages: (A) assessment of acute toxicity (LD50) for 1245 water-soluble organic lithium salts, (B) assessment of bioavailability (%) and pharmacokinetic parameters (Cmax, tmax, Vd), (C) assessment of various pharmacodynamic effects (IC50, EC50). At the first stage, 38 minimally toxic lithium salts (LD50 > 1000 mg/kg) were selected. At the second stage, the 11 lithium salts with maximum bioavailability (>20%): ascorbate, nicotinate, oxybutyrate, orotate, citrate, gluconate, comenat, pyroglutamate, glycinate, asparaginate, lactate were identified. At the third stage, various biological and pharmacological effects of the selected lithium salts were assessed and an analysis of possible interactions of the lithium ion with human proteome proteins was carried out. In general, the chemoreactomic analysis showed that lithium ascorbate is a remarkable candidate for further studies. Furthermore, we present some of the estimates of various properties of lithium ascorbate in comparison with the “control” salts (lithium nicotinate, lithium oxybutyrate, and lithium carbonate). These salts were chosen as examples because they are used in normothymic (mood stabilizer) drugs.

In comparison with the control substances, lithium ascorbate was characterized by more prominent inhibition of the reuptake of serotonin and dopamine and by a greater affinity for the inhibition of glutamate and α-adrenergic receptors. Lithium ascorbate can also be characterized by anti-inflammatory action (due to modulation of prostaglandin metabolism), to exhibit moderate anticoagulant, antihyperlipidemic, antihyperglycemic and antitumor effects (Table 1).

Lithium ascorbate is a highly effective antioxidant and, therefore, is metabolized and excreted from the body faster than the reference molecules. For lithium ascorbate, a lower T1/2 value in human liver microsomes (8.4 min, other molecules—more than 25 min) and a higher clearance value in liver microsomes (ascorbate—29 mL/min, nicotinate—19 mL/min, oxybutyrate—21 mL/min) were found.

It is important to note that lithium ascorbate may be more effective than nicotinate and oxybutyrate (see Table 1) in inhibiting the activity of the GSK-3β enzyme (ascorbate—47%, nicotinate—3.3%, oxybutyrate—6%)—one of the main target proteins of the lithium ions. The inhibition constant of GSK-3β was IC50 = 79 nM for lithium ascorbate and was much higher for the rest of the salts studied (IC50 = 1658–8240 nM). Let’s remind the reader that the lower values of an inhibition constant correspond to a higher inhibition of the corresponding target protein.

### 2.2. Neurocytological Studies of Lithium Salts on Cultured Cerebellar Granular Neurons

A glutamate stress model evaluated the synthesized lithium ascorbate substance in neurocytological studies on cultured cerebellar granular neurons (CGN). Studies have shown that lithium ascorbate was more effective in supporting neuronal survival than lithium chloride or lithium carbonate in the 0.1–1.0 mM concentration range. At the same time, for lithium chloride and for lithium carbonate, a significant dispersion of the values of neuronal survival was observed, which did not allow us to register any statistically significant differences when compared to the control.

**Figure 1:**
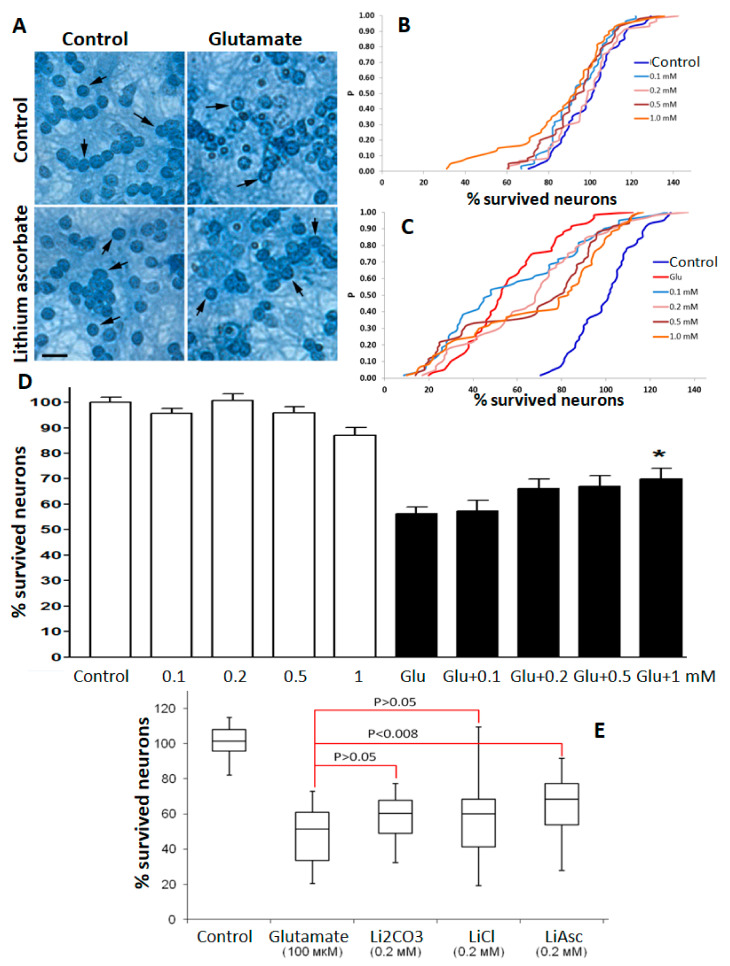
Results of neurocytological studies of lithium salts. (**A**) Representative photos of cultures of cerebellar granular neurons (indicated by arrows). Trypan blue staining of fixed cultures. Scale 1:0.000015. (**B**) Empirical distribution functions (e.d.f.) of the survival rate of neurons without the addition of glutamate (blank experiment); (**C**) E.d.f. of CGN survival under conditions of glutamate stress (100 μM glutamate). (**D**) Evaluation of the neuroprotective effect of lithium ascorbate according to the ANOVA test. White bars—lithium ascorbate (0.1–1.0 mM) in the absence of glutamate, black bars—at the same concentrations in the presence of 100 μM glutamate (Glu). K—control. For each column, 30 fields of view were calculated. *—*p* <0.01 compared with glutamate without the addition of drugs. (**E**) Comparison of the neuroprotective efficacy of various lithium salts (at a concentration of 0.2 mM) under conditions of moderate glutamate stress (100 μM glutamate, 50% neurons survived). Li_2_CO_3_, lithium carbonate; LiCl, lithium chloride; LiAsc, lithium ascorbate. P, statistical significance in accordance with the Kolmogorov–Smirnov test.

Following the analysis of the empirical distribution functions (e.d.f.) by the Kolmogorov–Smirnov method, the addition of lithium ascorbate in concentrations from 0.1 to 1 mM was non-toxic for CGN in the “blank” experiment (see Methods). Under conditions of glutamate stress, lithium ascorbate in concentrations of 0.2–1.0 mM significantly and dose-dependently increased the survival rate of CGN. The most pronounced neuroprotective effect was observed at lithium ascorbate concentration of 1 mM: neuronal survival increased, on average, by 11% (Kolmogorov’s maximum deviation D = 0.45, *p* < 0.001 according to the Kolmogorov–Smirnov test, Figure 1). The use of lithium ascorbate even at the minimum concentration (0.1 mM) led to significant differences (maximum Kolmogorov’s deviation D = 0.19; *p* = 0.049). With an increase in the concentration of lithium ascorbate, the significance of differences increased (the value of the maximum deviation D increased and the values of P decreased). An ANOVA test showed that neuronal survival under the cytotoxic effect of glutamate in the range of lithium ascorbate concentrations from 0.1 to 1 mM was significantly dose-dependently increased (on average, by 12%). At the same time, the use of a non-lithium salt of ascorbic acid (potassium ascorbate) was characterized by a much less pronounced neuroprotective effect: neuronal survival increased, on average, only by 5–6% (data not shown).

In accordance with the analysis of e.d.f. by the Kolmogorov–Smirnov method, under conditions of moderate glutamate stress, lithium carbonate at concentrations of 0.1–0.5 mM did not show a significant effect on the survival of CGN and at a concentration of 1 mM. On the contrary, Li_2_CO_3_ led to a significant decrease in neuronal survival (on average, by 25%, the maximum deviation D = 0.52, *p* < 0.0001). The latter result was confirmed by analyzing the survival rate of CGN using the Dunn test.

Under glutamate stress, lithium chloride at a concentration of 1 mM also led to a significant decrease in neuronal survival (on average, by 9%, maximum deviation D = 0.24, *p* < 0.005). Analysis of individual series of experiments using Dunn’s test showed that the protective effect of lithium chloride (16%, *p* < 0.05 at a concentration of 0.1 mM) manifested itself only in the first series of experiments and was not reproduced in five other series of experiments. The differences in the effects of the studied lithium salts under glutamate stress are confirmed by the results of a comparative analysis using the Kolmogorov–Smirnov test (Figure 1E).

### 2.3. Estimates of Lithium Biodistribution from Lithium Ascorbate Intake

A study of the compartmentalization of lithium in 11 biosubstrates of rats (brain, frontal lobe of the brain, heart, aorta, lungs, liver, kidneys, spleen, adrenal glands, femur, urine) was carried out after taking lithium ascorbate at a single dose of 1000 mg/kg. Within the mathematical framework of the non-compartmental analysis of the dynamics of concentrations in whole blood, we obtained the following values of the pharmacokinetic parameters of lithium ascorbate: Cmax = 50.59 μg/L, Tmax = 1.50 h, Clast = 33.7 μg/L, AUCt = 1750 μg/L∙h, MRTt = 22.9 h, Lz = 0.005 1/h, T1/2 = 141 h, CL = 0.029 l/h, Vd = 5.9 L.

**Figure 2:**
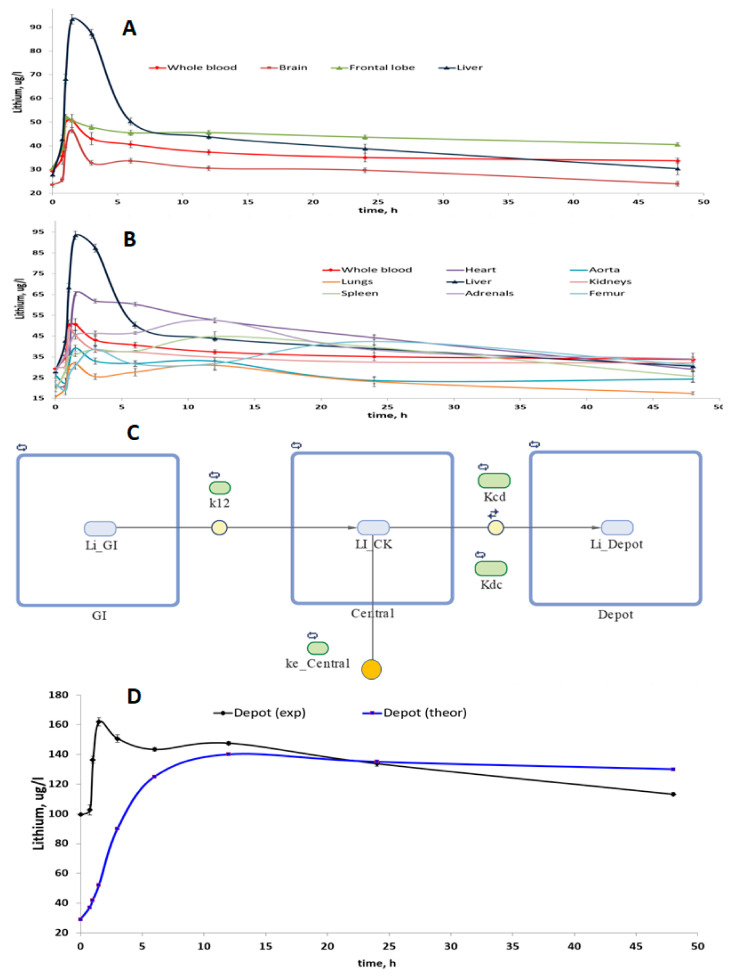
Analysis of lithium biodistribution after a single dose (1000 mg/kg) of lithium ascorbate. (**A**) Pharmacokinetic curves of lithium content in homogenates of brain tissue, whole blood and liver and, (**B**) other organs. (**C**) Three-chamber model of lithium ascorbate pharmacokinetics obtained as a result of multi-chamber pharmacokinetic modeling in MATLAB. The symbols for the corresponding constants are shown in the figure (k12, Kcd, Kdc, Ke_central). (**D**) Dynamics of concentrations in the “depot” of lithium are obtained due to multi-compartmental PK-modeling. The experimental data for the “depot” were obtained by summation of the lithium contents in the brain, aorta, adrenal glands, and femur.

The relevant pharmacokinetic curves (PK curves, i.e., the dependencies of concentrations on time) were obtained for homogenates of tissues of various organs (Figure 2A,B). Visual analysis of the PK curves showed that within 1–2 h after intake of lithium ascorbate, an intensive accumulation of lithium occurs in all tissues studied. The maximum values of the peak concentrations of lithium (Cmax) were observed for homogenates of liver and heart tissues and the minimum values of Cmax—in the lungs and aorta homogenates. It is important to note that the concentration of lithium in whole blood and in the frontal lobe of the brain remained stable for at least 40–45 h after passing the peak. 

This observation indicates first that the predominant accumulation of lithium in whole blood and in the frontal lobes when lithium ascorbate is used. Secondly, this observation indicates that the maintenance of lithium concentrations in these organs occurs by means of a certain “depot” of lithium. Indeed, multi-compartmental analysis confirmed that the stabilization of lithium levels in the blood is supported by a special “depot” of lithium (which, most likely, consists of the aorta, femur, and brain, Figure 2C,D).

One-, two-, three- and four-compartment models in various configurations were investigated. As a result of modeling, it was found that the simplest model that most accurately described the studied PK curves was a three-chamber model, which included the gastrointestinal tract (first compartment), whole blood (central, second compartment) and lithium depot (third compartment). Moreover, in this model of the best quality possible (for the data collected), the elimination of lithium was carried out from the central compartment and not from the depot or the first compartment (Figure 2C). The quality of the investigated multi-compartmental models was characterized by the values of the standard deviation of concentrations between the theoretical and experimentally obtained PK curves (a = 3.4 μg/L with a correlation coefficient of 0.92.

Simulations have shown that the volume of the depot can be approximately half the volume of the central compartment (i.e., of the whole blood). In this model, the lithium ascorbate is quickly transferred from the gastrointestinal tract to the blood (k12 = 0.67 1/h) and is very slowly removed from whole blood (which corresponds to a small value of the constant ke_Central = 0.0068 1/h, Figure 2C). The rate of lithium exchange between the blood and the depot is comparable to the rate of transfer from the gastrointestinal tract to the blood, and the transfer of lithium from whole blood to the depot (Kcd = 0.41 1/h) is somewhat faster than the reverse process of (Kdc = 0.27 1/h).

### 2.4. Acute and Chronic Toxicity Studies of Lithium Ascorbate

Toxicity studies indicated that lithium ascorbate is characterized by low acute and chronic toxicity. In acute toxicity studies with a single dose of 3000 mg/kg of lithium ascorbate, the lethality was 0%, and any detectable pathological changes were absent (not even a localized irritation). At 4000 mg/kg, the (delayed) mortality was 20%, intoxication manifested itself as depression, diarrhea, tousled coat, bloody discharge from the nose and eyes in males, and diarrhea in females. Pathological changes included hemorrhages of the membranes of the brain, edema and hemorrhages in the lungs.

Finally, for white rats of the Wistar line, the LD50 of lithium ascorbate was estimated to be 6334 mg/kg of body weight, and the LD100 was 8000 mg/kg. Thus, lithium ascorbate belongs to the 5th class of “practically non-toxic compounds”, LD50 ≥ 5000 mg/kg. Analysis of the results of determining the safety of lithium ascorbate by the Kerber and Pershin method for determining LD50 (Prozorovsky, 2007) showed that the differences do not exceed 0.1%. In comparison with lithium carbonate (LD50 = 531 mg/kg), lithium ascorbate was apparently 12 times less toxic.

In the study of chronic toxicity in Wistar rats, no teratogenic and embryotoxic effects of high doses of lithium ascorbate (1/10 LD50 per week) were observed in newborn rat pups. The cytogenetic effect of lithium ascorbate (according to the method of Zolotareva T.N.) led only to a decrease in mitosis though the difference between the control and the experimental groups was not significant.

The toxic effects of high doses of lithium ascorbate (1/10 LD50 daily during a month) on protein metabolism were absent. In particular, the intake of the lithium salts did not cause a decrease in the amount of essential amino acids. We also found that intake of lithium ascorbate did not cause a statistically significant effect on the decrease of the contents of free histidine, leucine, isoleucine, tyrosine, serine and methionine in the blood serum of rats. A trend (*p* = 0.06) was found for an increase in the content of linolenic, docosahexaenoic acids (increased by 15%), and dihomo-γ-linolenic acids (by 13%). The tendency to decrease the level of fatty acids was observed for oleic, pentadecanoic, palmitic acids (by 12–17%) and for myristic and arachidonic acids (by 5–7%). In brief, the results of toxicological studies indicate a good safety profile of the lithium ascorbate, even when used in high doses per os.

### 2.5. Assessment of the Antitumor Effects of Lithium Ascorbate

**Figure 3:**
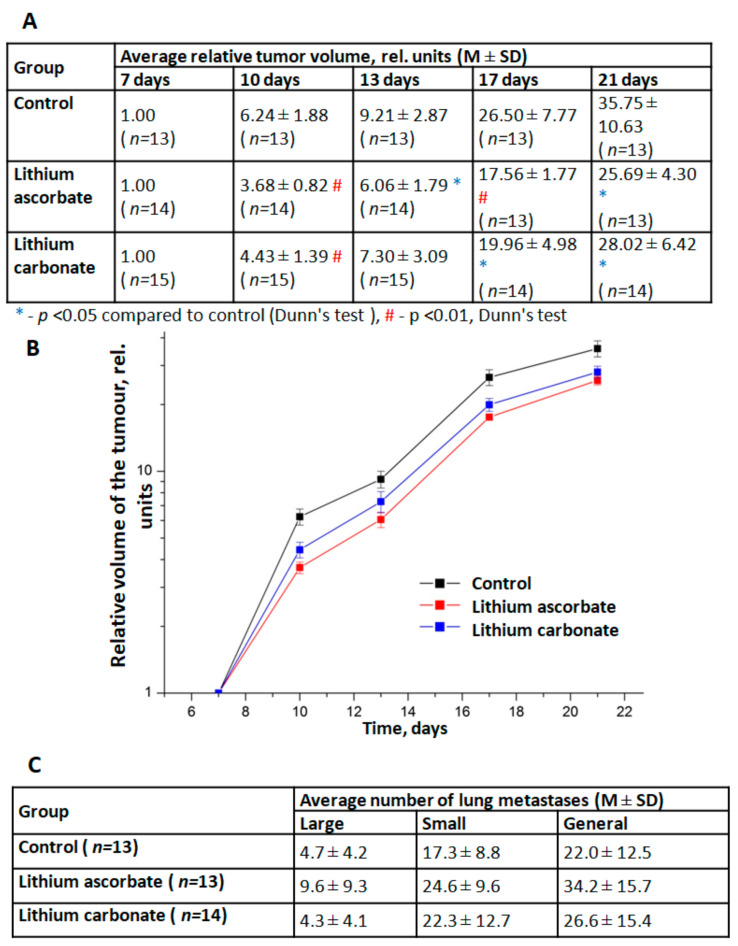
Assessment of the antitumor effects of lithium ascorbate and lithium carbonate. (**A**) Dynamics of the relative growth of Lewis lung carcinoma in the experimental groups. (**B**) Curves of relative LLC growth. (**C**) Calculation of the number of pulmonary metastases in the experimental groups on the 21st day of LLC growth.

Studies of the dynamics of growth and metastasis of Lewis lung carcinoma transplanted mice F1 (CBAxC57 Bl/6j) have shown that lithium ascorbate exhibits moderate anti-tumor effects. Two series of experiments were carried out. In the first series, a comparative study of the effects of different doses of lithium ascorbate (5 and 10 mg/kg) was carried out. In the second series, a comparison of the effects of lithium ascorbate and lithium carbonate was carried out for the same dose (5 mg/kg). Analysis of the dynamics of LLC growth in different groups showed that both studied drugs, already after 3 days from the start of their use, caused moderate (by 10–15%) inhibition of LLC growth in tumor-bearing animals (Figure 3A,B). At the same time, the effect of lithium ascorbate was more pronounced and stable: a statistically significant effect of this drug was noted from day 10 and throughout the entire observation period, and the TPO index was at a fairly high level (30–40%). The antitumor effect of lithium carbonate in this experiment was less pronounced and stable (TPO = 20–30%). The studied lithium salts did not have any significant effect on the processes of metastasis of LLC and the growth of lung metastases (Figure 3C).

### 2.6. Neuroprotective Properties of Lithium Ascorbate in the Model of Alcohol Intoxication

The effects of lithium ascorbate were investigated on a model of chronic alcohol intoxication, in which deviant behavior of animals is combined with irreversible degenerative changes in the liver and in the central nervous system (including demyelination of nerves). Lithium ascorbate at doses of 5, 10, and 30 mg/kg normalized behavioral responses in the open field and elevated plus-maze tests. An increase in the dose of lithium ascorbate (10 mg/kg, 30 mg/kg) did not lead to a significant improvement in the studied parameters of the state. Histological analysis showed that the use of lithium ascorbate minimized the level of ischemic damage to neurocytes to the level of a reversible state and promoted the preservation of the myelin sheaths of the nerves.

When the model of alcohol intoxication was reproduced, behavioral reactions were disturbed (chaotic movement without manifestations of exploratory and search behaviors). Histological analysis showed circulatory disorders, which were characterized by hemostasis in the capillaries and venules with the development of severe perivascular edema of the nervous tissue. Toxic damage to neurocytes in the cerebral cortex was characterized by acute swelling of the pyramidal cells with rounding of the cell body, swelling of the axon, homogenization of the cytoplasm with the disappearance of Nissl granulations, and disruption of the contours of the nucleus.

**Figure 4:**
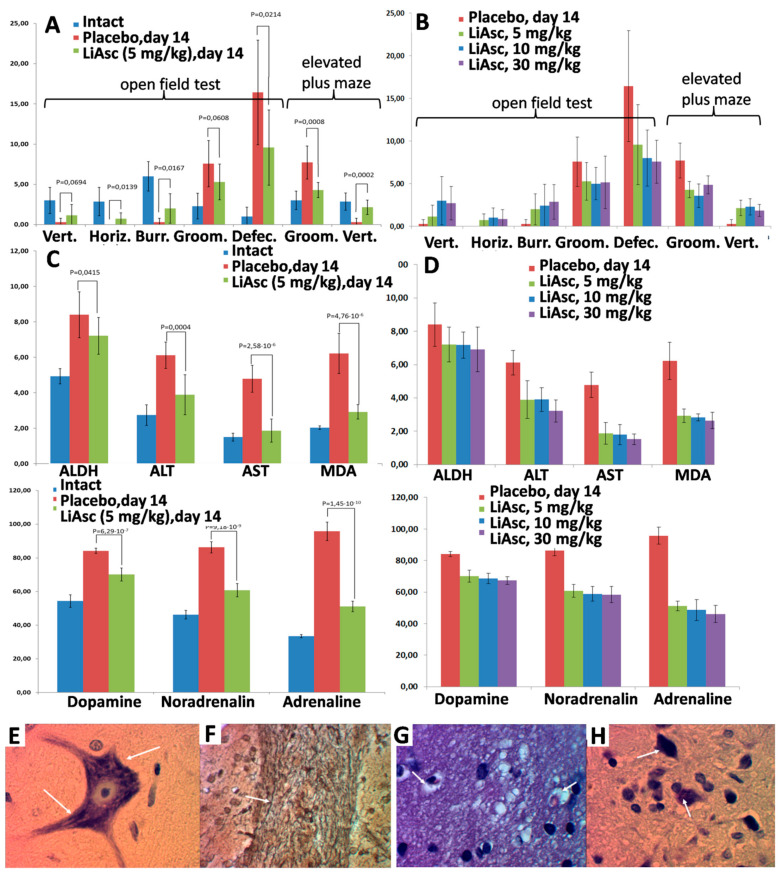
Effects of “prophylactic” use of lithium ascorbate in animals with the model of alcohol intoxication. (**A**) Indicators of neurological tests, 5 mg/kg. (**B**) Dose-dependence, neurological tests. (**C**) Biochemical indicators. (**D**) Dose-dependent effects on biochemistry. (**E**) Histological effects of lithium ascorbate. Focal fusion of Nissl lumps in the cytoplasm of the pyramidal cell. Nissl staining with toluidine blue. Magnification ×1200. (**F**) Clear contrasted contours of the commissural fibers of the brain with preservation of the integrity of the myelin sheath. Silver impregnation. Increase ×1200. (**G**) Histological picture. Pronounced pericapillary edema of the nervous tissue. Staining with hematoxylin and eosin. Magnification ×1200. (**H**) Hyperchromatosis, pycnosis of neurocytes. Astrogliocyte hypertrophy. Nissl staining with toluidine blue. Magnification ×1200.

The “prophylactic” use of lithium ascorbate in alcohol intoxication improved vertical activity and other indicators of neurological tests (Figure 4A,B). Long-term alcohol intoxication caused an increase in the concentration of catecholamines in the blood, and the use of lithium ascorbate prevented an increase in the content of catecholamines in the blood of animals. The greater efficiency of lithium ascorbate was also manifested by the activation of ADH in the liver, which corresponds to the acceleration of ethanol elimination (Figure 4C,D). Alcohol caused a significant increase in serum MDA, while lithium ascorbate reduced MDA levels (which corresponds to inhibition of lipid peroxidation and activation of the body’s antioxidant system). Higher doses did not lead to a significant increase in the effectiveness of lithium ascorbate (Figure 4B,D).

The “prophylactic use” of lithium ascorbate under conditions of chronic alcohol intoxication significantly alleviated the histological manifestations of cerebral ischemia. Compared with the placebo group, in all groups of animals receiving lithium ascorbate, circulatory disorders of the nervous tissue were characterized by focal hemostasis in the capillaries and moderately pronounced pericapillary edema of the nervous tissue, the cortex, the white matter of the cerebral hemispheres, and the brain stem.

When lithium ascorbate was taken, a significant part of the neurocytes of the cortex and subcortical nuclei were characterized by reversible changes, which were expressed by dispersion and indistinctness of the tigroid contours, the focal fusion of Nissl lumps in the cytoplasm, moderately pronounced swelling of the nucleus and axonal process. The macroglial reaction of the nervous tissue was minimal and was expressed by edema of the periarteriolar astrocytes. The impregnation of the pathways of the brain with silver showed the preservation of the myelin sheaths of the nerve fibers, which had clear contours (Figure 4E–H). Morphometric analysis showed that in the group of animals receiving lithium ascorbate at a dose of 5 mg/kg, the number of damaged nerve cells in the cortex was 18.5%, and in the placebo group, the number of damaged cells was 34.8% (with significant morphological signs of irreversible death of neurocytes). Higher doses of lithium ascorbate did not lead to a substantial improvement of the results of morphometric analysis.

In the “therapeutic” series of experiments, lithium ascorbate (5, 10, 30 mg/kg) was administered immediately after the alcohol solution was discontinued. After introducing the lithium ascorbate, the number of aggressive incidents was reduced. The animals showed a more profound and longer sleep, fell intoa stupor less often, and quickly adapted to the absence of alcohol in the diet. The histological analysis showed that lithium ascorbate as compared to placebo contributed to a reduction in circulatory disorders of the nervous tissue and focal hemostasis in the capillaries, to a less pronounced pericapillary edema of the nervous tissue in the cortex, to greater preservation of the white matter of the cerebral hemispheres and the brain stem. 

In the groups on lithium ascorbate, only single neurocytes with slight injuries were noted. These injuries included the indistinctness of the tigroid contours, the focal fusion of Nissl lumps in the cytoplasm, and moderately pronounced swelling of the nucleus. The state of the conduction system of the brain of animals receiving placebo was characterized by a greater focal swelling of myelin fibers with an uneven distribution of myelin, which, when impregnated with silver, created a picture of fuzzy contours. Structural changes in the brain’s nerve fibers in the group taking 5 mg/kg lithium ascorbate were limited to local edema of the nerve fibers with complete preservation of the myelin sheath. Higher doses of lithium salt did not improve the histological picture. In general, both “prophylactic” and “therapeutic” use of lithium ascorbate contributed to the relief of withdrawal symptoms and prevented formation of irreversible degenerative changes of the nervous tissue.

## 3. Discussion

The present study combines the use of modern methods of data mining/artificial intelligence (chemoreactomic analysis) with experimental studies of the most promising candidates of lithium salts based on anions of organic acids. **Chemoreactomic modeling** showed that lithium ascorbate is a remarkable candidate for further studies.In comparison with the control substances, lithium ascorbate was characterized by more prominent inhibition of the reuptake of serotonin and of dopamine and by a greater affinity for the inhibition of glutamate and α-adrenergic receptors. It is important to note that lithium ascorbate may be more effective than nicotinate and oxybutyrate (see Table 1) in inhibiting the activity of the GSK-3β enzyme (ascorbate—47%, nicotinate—3.3%, oxybutyrate—6%)—one of the main target proteins of the lithium ions. The inhibition constant of GSK-3β wasIC50 = 79 nM for lithium ascorbate and was much higher for the rest of the salts studied (IC50 = 1658–8240 nM).

Chemoreactomic modeling also indicated a lower affinity of lithium ascorbate for the potassium channel KCNH2 (Table 1). This potassium channel is an important “anti-targeting” protein interaction that should be avoided during drug development (since the disruption of KCNH2 activity can lead to the dangerous “long QT syndrome”). Prediction of the properties of lithium ascorbate showed that when taking doses of about 10 mg/kg/day, there will be no significant change in hematocrit (which is associated with changes in the water–salt balance of blood cells). Changes in hematocrit estimated for nicotinate and oxybutyrate lithium salts were apparently higher.

It is also important to notice that chemoreactomic assessments of the probability of interaction of the studied molecules with various transporter proteins suggested that lithium ascorbate can enter cells by means of SLC23A1, SLC23A2 transporters (vitamin C transporter proteins) [14,15]. The probabilities of the interaction of lithium ascorbate with these proteins were 0.9–0.95 a.u. and were much lower for the rest of the molecules (0.1–0.4 a.u.). Since these transporter proteins are present in neurons, the ascorbate anion in lithium ascorbate increases the bioavailability of the lithium ions. It promotes an increase in the accumulation of Li+ in the nervous tissue. Thus, lithium ascorbate represents a promising lithium compound for analyzing neuroprotective properties.

The results of **neurocytological studies** indicate a direct neuroprotective effect of lithium ascorbate on cerebellar granular neurons in culture. Treatment of neurons in culture with lithium ascorbate increased cell survival under conditions of glutamate stress, and this effect was almost indistinguishable for the inorganic lithium salts (carbonate, chloride) in the same range of concentrations (0.1–1.0 mM).

The studies of **lithium biodistribution** were performed using the standard math models of pharmacokinetic analysis. The biodistribution data collected herein (groups of animals taken at certain time periods) considerably differ from the classical pharmacokinetic study (samples of blood taken at certain time periods). Nevertheless, the usage of the pharmacokinetic models allowed us to describe quantitatively the peculiarities of the biodistribution of lithium in rats.

Pharmacokinetic-based math modeling has shown that the volume of the lithium depot can be approximately half the volume of the central compartment (i.e., of the whole blood, Figure 2). Lithium (ascorbate) is quickly transferred from the gastrointestinal tract to the blood (k12 = 0.67 1/h) and is very slowly removed from whole blood (which corresponds to a small value of the constant ke_Central = 0.0068 1/h, Figure 2C). The rate of lithium exchange between the blood and the depot is comparable to the rate of transfer from the gastrointestinal tract to the blood, and the transfer of lithium from whole blood to the depot (Kcd = 0.41 1/h) is somewhat faster than the reverse process of lithium transfer from the depot to the whole blood (Kdc = 0.27 1/h).

Comparison of the dynamics of the concentration of lithium in the depot, obtained as a result of the simulation, with the dynamics of the concentration of lithium in the “depot”, consisting of the brain, aorta, adrenal glands, and femur, indicates certain similarities in changes in concentrations. Obviously, the depot, consisting of these organs, allows for the stabilization of lithium concentrations after the first 10–15 h of the experiment. Thus, lithium ascorbate contributes to the maintenance of stable concentrations of lithium ions in the blood and in the brain, which is important for the implementation of the preventive and therapeutic potential of the lithium ions.

The results of **toxicological studies** indicate a good safety profile of the lithium ascorbate, even when used in high doses. For white rats of the Wistar line, the LD50 of lithium ascorbate was estimated to be 6334 mg/kg of body weight, and the LD100 was 8000 mg/kg, indicating extremely low toxicity.

The mice model of lung Lewis carcinoma (LLC) studies indicated the onco-safety of lithium ascorbate and shown even a small but significant **antitumour effect**. Analysis of the dynamics of LLC growth in different groups showed that both lithium salts, ascorbate, and carbonate, after 3 days from the start of their use, caused moderate (by 10–15%) inhibition of LLC growth in tumor-bearing animals (Figure 3). At the same time, the effect of lithium ascorbate was more pronounced and stable: a statistically significant effect of this drug was noted from day 10 and throughout the entire observation period while the TPO index of tumor growth inhibition was at a fairly high level (30–40%). The antitumor effect of lithium carbonate in this experiment was less pronounced and stable (TPO = 20–30%). The studied lithium salts did not have any significant effect on the processes of metastasis of LLC and the growth of lung metastases.

The neurological effects of lithium ascorbate were investigated on a **model of chronic alcohol intoxication**. The deviant behavior of animals is combined with irreversible degenerative changes in the liver and central nervous system (including demyelination of nerves). Studies on the model of alcohol intoxication in rats have shown that intake of lithium ascorbate in doses either 5, 10 or 30 mg/kg reduced brain damage due to ischemia and also ramped up preservation of myelin sheaths of neurons.

In both “prophylactic” and “therapeutic” series of experiments **morphometric analysis** showed that in the group of animals receiving lithium ascorbate at a dose of 5 mg/kg, the number of damaged nerve cells in the cortex was 18.5%, and in the placebo group, the number of damaged cells was 34.8%, with significant morphological signs of irreversible death of neurocytes. The higher doses of lithium ascorbate did not result in any significant improvement in the results of morphometric analysis.

## 4. Materials and Methods

The complex of studies included in silico chemoreactomic modeling of lithium salts, synthesis of lithium salt(s), neurocytological studies, assessments of biodistribution, studies of acute and chronic toxicity, assessment of antitumor effects, adaptogenic, stress-protective and neuroprotective properties of lithium ascorbate on the model of alcohol intoxication.

During the animal studies, the animals were kept under standard conditions in accordance with Directive 2010/63/EU of the European Parliament and of the Council of the European Union of 22 September 2010 concerning the protection of animals used in scientific studies [16]. Indoor air control in compliance with environmental parameters (temperature 18–26 °C, humidity 46–65%). The rats were kept in standard plastic cages with bedding; the cages were covered with steel lattice covers with a stern recess. The floor area per animal met regulatory standards. The animals were fed in accordance with Directive 2010/63/EU. The animals were given water *ad libitum*. The water was purified and normalized for organoleptic properties in terms of pH, dry residue, reducing substances, carbon dioxide, nitrates and nitrites, ammonia, chlorides, sulfates, calcium and heavy metals in standard drinkers with steel spout lids.

**Chemoreactomic modeling of lithium salts.** The chemoreactomic approach to the analysis of the “structure-property” problem of molecules is the newest direction of the application of artificial intelligence systems in the field of post-genomic pharmacology. The methodology and the algorithms realized in the original proprietary software developed by the authors were described [12,13] and tested [15]. The training of the algorithms of metric analysis [15] was carried out on the basis of the data on structure and properties of the molecules presented in the PubChem, HMDB, STRING databases. The algorithms and software developed were realized within the framework of the topological theory of data analysis as described earlier [12]. The algorithms that predict molecular properties were validated using cross-validation estimates as described in [13]. Based on the algorithms developed, we estimated over 350 pharmacological properties of lithium salts relevant to their toxicity, pharmacokinetics, pharmacodynamics, etc. The list of the lithium salts studied (water-soluble organic lithium compounds with a molecular weight of less than 300 Da) along with the structural formulas was downloaded from the PubChem database (totally, 1245 compounds encoded in SMILES format). The algorithms for predicting molecular properties were extensively tested earlier on over 100,000 molecules [12,15]. Additional information about the algorithms is presented available online at www.chemoinformatics.ru and www.pharmacoinformatics.ru (both sites were accessed on 21 February 2022).

**Synthesis of lithium salts**. The synthesis of the lithium salts was based on the classical reaction of neutralization carried out with the organic acids and the lithium carbonate. A detailed description of the synthesis of lithium ascorbate is presented below. The synthesis was carried out in accordance with the equation of the reaction of neutralization 2C_6_H_8_O_6_ + Li_2_CO_3_ = 2C_6_H_7_O_6_Li + H_2_O + CO_2_↑. To a suspension of 17.6 g (0.1 M) of ascorbic acid (L-Ascorbic acid C_6_H_8_O_6_, LLC Sigma-Aldrich Rus, Moscow, Russia, A5960, BioXtra, ≥99.0%) in 25 mL of distilled water we added the lithium carbonate (lithium carbonate Li₂CO₃, Merck KGaA, Moscow, Russia, 1.05671.1000, certificates Ph Eur, BP, USP) in the amount of 3.7 g (0.05 M). Li₂CO₃ was added in portions of 0.5 g with constant stirring and heating to 35–45 °C. Each successive portion of lithium carbonate was added only after termination of the copious production of the CO_2_ gas. After adding the last portion of lithium carbonate, the suspension was heated to 70–80 °C until complete dissolution of the remaining mix of ascorbic acid and the lithium carbonate. As the result of neutralization, a clear, pale, straw-colored solution with pH≈6–7 was obtained. When the solution was cooled to room temperature in 30–50 min, an abundant crystalline precipitate formed, which was filtered and washed with ethanol. The alcohol wash was combined with the filtrate in the 1:2 ratio and then placed into cold storage (+4 °C) overnight. The precipitate formed in cold storage was filtered, washed with a small amount of alcohol, and combined with the precipitate obtained earlier. The synthesized lithium ascorbate was dried in a vacuum oven at 30–50 °C. The total yield of lithium ascorbate was 80–90% (14–16 g).

**Neurocytological studies of lithium salts on a culture of cerebellar granular neurons.** We used seven-day cultures obtained by the method of enzymatic-mechanical dissociation of cerebellar cells of 7-day-old rats as described previously [17]. Cultivation was carried out in 96-well plastic plates for 7 days in a CO_2_ incubator filled with gas mixture (95% air + 5% CO_2_) at the temperature of 35.5 °C and the relative humidity of 98%. Solutions of lithium salts were prepared from dry anhydrous salts. The inorganic lithium salts were manufactured by Merck KGaA, Moscow, Russia (Lithium carbonate Li₂CO₃, 1.05671.1000, Ph Eur, BP, USP certifications; Lithium chloride LiCl, 1.05679.0100, Reag. Ph Eur certifications), the organic salts—as described above. Dry salts were dissolved in deionized water at a concentration of 10 mM, then sterilized by ultrafiltration and added to the cultivation medium on day 2 in vitro for the entire period of cultivation (up to 7 days).

The state of the neuronal cultures was monitored daily by visual inspection under microscope with phase contrast. The final concentrations of the lithium salts in the culture medium were 0.1, 0.2, 0.5 and 1 mM. The experimental design used was described earlier [4]. Briefly, monosodium glutamate (L-Glutamic acid monosodium salt 99–100%, LLC Sigma-Aldrich Rus, Moscow, Russia, NG-1626) was added to cultures at three different concentrations (50, 100 and 150 μM) to estimate the toxic effect. In this series of experiments the optimal glutamate concentration was 100 μM and it corresponded to the moderate-to-mild neurocytotoxic effect of glutamate (survival 30–70% of the neurons in control group which allowed us to assess the neuroprotective properties of the substances studied). The test substances were then introduced into the cultivation medium on the second day and left until the seventh day.

The study of the effects of each salt was carried out in two stages: (1) the “blank” experiment, in which the effects of different concentrations of lithium salt on the survival of neurons were studiedwithout addition of glutamate, and (2) the “main” experiment, in which neurons were cultured under conditions of glutamate stress at different concentrations of lithium salts and the survival rates of the cultured neurons were measured.

The quantitative assessment of neuron survival was performed using direct counting of neurons with unchanged morphology [17]. For each salt, five experiments were performed; for each concentration of a salt at least three cultures were taken, each of which was photographed and counted using three consecutive visual fields. The number of neurons with unchanged morphology in control cultures was taken to be 100%. The ANOVA test with Bonferroni correction and the Dunn test were used for statistical analysis. Additionally, the analysis of empirical distribution functions (e.d.f.) of neuron survival values was carried out using the statistical test of Kolmogorov–Smirnov. The differences between groups were considered to be significant at *p* < 0.05 level, the results were expressed as mean ± standard deviation.

**Estimates of the biodistribution of lithium after intake of lithium ascorbate**. The experiments were carried out on male white Wistar rats (200–250 g, nine groups of six animals each). The animals were probed with solution of lithium ascorbate, 1 mL of which contained 250 μg of elemental lithium. The solutions were prepared using anhydrous powder of lithium ascorbate. The required volume of solution was calculated based on the weight of the animal in order to reach 1000 μg/kg dose of elemental lithium. After the probing with a solution of lithium ascorbate the biosubstrates studied (whole blood, brain, frontal lobe of the brain, heart, aorta, lungs, liver, kidneys, spleen, adrenal glands, femur) were taken successively from each of the 9 groups of animals at the nine time points (0 min, 45 min, 1 h, 1.5 h, 3 h, 6 h, 12 h, 24 h, and 48 h).

The lithium levels in homogenates of these 11 different biosubstrates were determined using mass spectrometry. The samples of homogenates were taken into plastic tubes and diluted 5 times with bidistilled and deionized water. The internal standard (indium at a concentration of 25 μg/L) was added to the solutions. Calibration solutions were prepared from VTRC standard solutions with a known content in the range from 5–1000 mgc/L. The resulting solutions were analyzed on inductively coupled plasma ionization mass spectrometer “Plasma Quad PQ2 Turbo” (VG Elemental, Winsford, Cheshire, UK). The power of the microwave generator was 1.3 kW, the flow rate of the plasma gas (argon) was 14 L/min, and the flow rate of the carrier gas was 0.89 mL/min. From 3 to 10 exposures of each sample were carried out; the signal integration time was 60 s.

The methods of multi-compartmental and non-compartmental pharmacokinetic analysis were used to quantify biodistribution of lithium. The multi-compartmental analysis was carried out using the SimBiology package as part of the MATLAB2016 software package [18], and the non-compartmental analysis was performed using Excel spreadsheets, supplemented by the modules of the PKSolver software package [19].

**Study of acute and chronic toxicity of lithium ascorbate.** The experiments were carried out on male and female Wistarrats 8 weeks of age at various concentrations of lithium ascorbate (0, 3000, 4000, 5000, 6000 and 7000 mg/kg). The total number of groups was 6, each group contained 10 rats (5 males 205.8 ± 1.89 g and 5 females 174.4 ± 1.66 g). Lithium ascorbate was administered intragastrically. In the study of acute toxicity, the tested and the control substances were taken once (fractionally, using a probe) with further observation of the animals for 14 days. Animals were deprived of food for 16 h before administration of the substances, before bodyweight recording and before euthanasia. Access to water was not restricted throughout the experiment. The cytogenetic effects were estimated after T.N. Zolotareva’s methodology [20].

The animals were under continuous observation before administration of the lithium salt, within 30 min after the administration of the last portion of the salt, then hourly for 4 h, then after 24 h and then daily for 15 days. Behavior (depression/excitement), reactions to stimuli, condition of the skin and mucous membranes, secretions, muscle tone, coordination of movements were registered. Clinical examination of the animals was carried out before administration, then on the second, eighth and fourteenth days of the experiment. For histological examination, the material was fixed in a 10% solution of neutral formalin for 24 h, after which it was embedded in paraffin according to the generally accepted technique. Then sections with a thickness of 5–7 μm were made, which were stained with hematoxylin and eosin. The calculation of LD50 was carried out using the Bliss–Prozorovsky method [21]. Statistical analysis was performed using the Statistica 10.0 software (StatSoft, Tulsa, OK, USA).

**Assessment of the antitumor effects of lithium ascorbate.** The study was performed on 42 male mice hybrids F1 (CBA×C57 BL6j) at the age of 2.5–3 months, body weight 23–26 g. The animals had a veterinary certificate and passed the 20-day quarantine in vivarium. The studies were carried out on transplantable Lewis lung carcinoma (LLC). The LLC strain was obtained from the bank of tumor materials of the N.N. Blokhin’s Institute and was maintained in male C57 BL6j mice. Transplantation of LLC to the male mice was carried out by subcutaneous administration of 1.9 × 10^6^ tumor cells in 0.1 mL of the suspension in the region of the lateral surface of the right thigh after depilation of the area.

The animals were included in the experiment on the 7th day after LLC inoculation, when the tumor node had already formed and reached a measurable size in all mice. The animals were randomized into three groups—control (13 mice) and two experimental groups for each of the lithium salts (14 mice). The animals of the control group did not receive any further treatment. From 7 to 20 days after LLC transplantation, lithium salts carbonate in doses of 5 mg/kg (elemental lithium) in 1% starch gel, which was made *ex tempore*, were given through the probe to the animals of the experimental groups from 7 to 20 days after LLC transplantation.

Tolerability of the studied lithium preparations was assessed by daily observation of animals, in which the neurological status was studied according to the peculiarities of spontaneous locomotor activity, general excitability and response to tactile and sound stimuli, as well as by the food activity of animals and the dynamics of their body weight gain.

The effect of lithium preparations on the tumor process was studied by the dynamics of growth and the activity of metastasis of LLC. To do this, two diameters of tumor nodes were measured by a caliperevery 3–4 days: *L*—the maximum diameter of the node; *W*—the diameter orthogonal to *L*. The calculation of the volumes of the nodes was carried out using the approximation $V=\left(L\times W^{2}\right)\times \left(\pi /6\right)$ that was shown to correlate reliably with the MRI data [22]. The effect of drugs on the growth of LLC was assessed by comparing the volume of tumor nodes in the control and experimental groups at different periods of observation, and then by calculating the growth inhibition index *TPO*: $\mathrm{TPO}=\left(V_{K}\;V_{O}\right)/V_{K}\times 100\%$, where $V_{K}$ and $V_{O}$ are the average volumes of nodes in the control and experimental groups, respectively. On the 21st day of carcinoma growth, the animals were removed from the experiment by cervical dislocation under ether anesthesia, the lungs were isolated, fixed for 24 h in Bouin’s fluid, and then the numbers of large and small lung metastases was counted. The effect of lithium preparations on the processes of metastasis and the growth of metastases was assessed by comparing the numbers of metastases in the control and the experimental groups. Intergroup differences were assessed by Kruskal-Wallis rank analysis of variance using Dunn’s test. Differences were considered significant at the 0.05 significance level. The calculations were performed using the Statistica 10.0 software package (StatSoft Inc., Tulsa, OK, USA).

**Alcohol intoxication model of neural damage.** The study was carried out on male white Wistar rats weighing 200–250 g (*n* = 168) as described in the guideline [23]. In brief, the selection criterion for rats, apart from absence of visible abnormalities in physical state and behavior, was the initial preference of 6% ethanol solution over the drinking water. To reveal this preference, a preliminary experiment was carried out for 3 days in individual cages with free access to both fluids. After selection, a 6% solution of ethyl alcohol was proposed as the only source of liquid; after a week, the concentration of alcohol was increased to 15%. After 2 weeks, the alcohol was replaced with drinking water.

The experiment was carried out in two series—“prevention” and “treatment”, four groups in each series: (1) dose of 30 mg/kg, (2) a dose of 10 mg/kg, (3) 5 mg/kg dose, (4) placebo group. In a series of “prevention” experiments, the drug was administered in parallel with the initial intake of alcohol and in a series of “treatment” experiments—after the reproduction of the model of alcohol intoxication. Each group consisted of 21 animals. At 14th day, the lithium salt was administered intragastrically through the probe, once a day in a volume of 0.5 mL for each animal.

A general assessment of the somatic and neurological status of animals included the estimates of the level of anxiety and other negative impacts of alcohol intake, the behavior of animals in the open field test and in the “elevated plus” maze test.

In the open field test (OFT), vertical locomotor activity, horizontal locomotor activity, the number of peeping into the holes (“burrow reflex”), the number of grooming acts, and the number of exits to the central zone were recorded. In the OFT test, the animal was placed in the same square located near the wall. The exposure time of each animal was 5 min. The round OFT involved an arena 1 m in diameter with a wall height of 0.4 m, the bottom of which was divided into sectors. In the open field, 3 zones were outlined: central, intermediate (6 sectors), peripheral (12 sectors). Lighting was provided by 2 lamps, 60 W each, which were located at a height of 1.5 m from the bottom of the chamber above the central segments of the field. The test recorded horizontal activity in the central and peripheral zones, vertical activity, the number of grooming acts, the number of defecation acts, etc. After each animal, the walls and bottom were treated with a wet and dry napkin.

In the “elevated plus” maze (EPM) test the setup used two arms, at the intersection of which there was an open area. One of the labyrinth arms had closed compartments. The labyrinth was installed at the height of 1 m from the floor. The stay of animals in the covered and closed arms and at the center of the labyrinth, the duration of grooming in the closed arms, number of grooming episodes in a closed arm were recorded. The time of testing animals in EPM was 5 min.

On days 0 and 14, the eight biochemical parameters (alanine transaminase ALT, aspartate aminotransferase AST, malondialdehyde MDA, dopamine, norepinephrine, adrenaline, serotonin in blood serum and alcohol dehydrogenase ADH in liver cells) were determined in all groups of animals. Blood sampling was performed from the sublingual vein by cutting the sublingual frenum. The blood contents were analyzed on an automatic biochemical analyzer “Konelab-20i” (Finland). Liver cells for determination of alcohol dehydrogenase were collected from animals after the experiment. ADH activity in hepatocytes was determined photometrically.

ALT and AST (μmol/mL/h) activities were determined on an automatic photometric analyzer ChemWell2910C (Palm City, FL, USA) using standard kits ALT-UTS and AST-UTS, respectively, produced by Eiliton LLC (Dubna, Russia). The metabolites MDA (nmol/mL) and AAD (nmol/mg protein/min) were measured on NanoDrop ™ 2000 microspectrophotometer. Serotonin was determined by the Michel’s method. Catecholamines (nmol/mL) were determined on a Waters 590 liquid chromatograph with an amperometric detector (NPO Khimavtomatika, Moscow, Russia) (working electrode material—glassy carbon) using an Ascentic C18 column (5 μm, 4.6 × 250 mm). Electrophoretic determination was carried out on a capillary electrophoresis system,“Kapel-105” (OOO Lumex, Saint Petersburg, Russia), with a spectrophotometric detector, an unmodified quartz capillary with a total length of 60 cm, an effective length of 50 cm, and an inner diameter of 50 μm.

**Histopathological analysis.** After craniotomy, the brain was removed and fixed in a 10% solution of neutral formalin. After 1 day, the zone of the precentral gyrus of the forebrain was isolated using frontal incisions. The nerve tissue was processed according to the standard scheme (dehydration in ethyl alcohol, xylene), followed by the manufacture of the paraffin blocks. Histological sections 5–6 µm thick were prepared on a microtome “Microm” and were stained with hematoxylin and eosin. Duplicate sections were stained according to the Nissl method and impregnated with silver using Biovitrum (Saint Petersburg, Russia) reagent kit. The morphometric study of histological sections was carried out on image analyzer BioVision GmbH (Vienna, Austria) and consisted of counting the damaged neurocytes of the pyramidal layer of the cerebral cortex in 10 different fields of view with subsequent statistical processing of the results. Micrographs were obtained using a Micros research microscope and a DCM 900 digital eyepiece camera (Oplenic Optronics Equipment Co., Zhejiang, China). The results were processed using the Excel 2013 and Statistica 10.0 (Tulsa, OK, USA) software packages. The significance of differences between groups was determined by a nonparametric U-test, the Wilcoxon–Mann–Whitney test.

## 5. Conclusions

Lithium ions have a significant effect on the homeostasis of acetylcholine, enkephalins, catecholamines, serotonin and exhibit neuroprotective properties. The toxic properties of the lithium carbonate drive the search for new salts to be used in lithium therapy. As shown by the results of this study, lithium ascorbate is a highly digestible and low toxicity lithium salt. A neurocytological study on cerebellar granular neurons in culture using a glutamate stress model showed that lithium ascorbate was more effective in supporting neuronal survival (+11%) than lithium chloride or carbonate. Estimates of the biodistribution of lithium have shown that when lithium ascorbate stimulates the accumulation of lithium ions mainly in the brain. Lithium ascorbate is characterized by extremely low acute and chronic toxicity and exhibits moderate antitumor effects. Studies in rats have shown adaptogenic and neuroprotective properties of relatively low doses of lithium ascorbate (5 mg/kg, which corresponds to ~1/1300 of LD50 of this substance).

## 6. Patents

A part of this work resulted in a patent: “Means with anti-stress, anxiolytic and antidepressant activity and composition based on it”, RU2617512C1, https://patents.google.com/patent/RU2617512C1/ru (accessed on 21 February 2022).

## Figures and Tables

**Table 1:** Results of chemoreactomic analysis of the lithium salts. “Const.”, The common name for the type of investigated pharmacological constant (“Ki”, “IC50”, etc.); “Error”—the error of the estimated biological constant; “Unit”, units of measurement.

Const.	Ascorbate	Nicotinate	Oxybutyrate	Carbonate	Error	Unit	Activity
Ki	648	2980	1503	8850	572	nM	Inhibition of serotonin reuptake
Ki	126	421	285	5450	559	nM	Inhibition of dopamine reuptake
Ki	622	1866	930	7402	320	nM	Inhibition of currents of N-methyl-D-aspartate glutamate receptors of the NR1a/NR2D type
Ki	173	438.7	862	6820	182	nM	Affinity for alpha-2 adrenergic receptors of rat brain homogenates, ligand
Ki	115	1838		5928	2539	nM	Displacement of N-methylscopolamine from M1 muscarinic receptors
IC50	79	2534	1658	8240	191	nM	Inhibition of GSK-3β
EC50	60.4	111.1		-	82	mg/kg	Modeling anticonvulsant activity in a model of electroshock in mice
-	3.8	37.4	8.83	29.1	4.8	%	Percentage change in hematocrit at a dose of 10 mg/kg/day
-	17.2	12.9	eight	-	1.9	%	Fraction of unbound substance in human blood plasma
T1/2	8.4	54	25	-	4	Min	Metabolic stability in human liver microsomes
CL	29	19	21	-	16	mL/min	Characteristic clearance in human liver microsomes
Ki	5308	1868	2995	-	2118	nM	Affinity for the K-channel KCNH2
IC50	5069	2746	1545	-	1199	nM	Inhibition of the K-channel KCNH2
IC50	113.9	4967	495	9130	351	nM	Inhibition of MMP-2
Ki	165.3	347.5	344	10,239	54	nM	Inhibition of MMP-9
-	41.25	22.16	25.1	-	34	%	Anti-inflammatory activity as inhibition of carrageenan-induced paw edema (25 mg/kg orally)
IC50	125.7	383	598	>40,000	507	nM	Inhibition of clotting factor F10
EC50	126	2264	1987	82,303	295	nM	PPAR delta agonist
-	14.98	8.08		0	5.5	%	Antihyperglycemic activity in rats as a decrease in blood glucose at a dose of 100 mg/kg, orally
IC50	185.5	3842	1702	>20,000	929	nM	Antiproliferative Activity Against Human QG56 cells
IC50	110.8	3096	1100	>20,000	242	nM	Antiproliferative activity against human H460 cells

## Data Availability

The data are available upon reasonable request.

## Sample Availability

Samples of the lithium ascorbate are available from the authors.
